# Proliferative diabetic retinopathy and relations among antioxidant activity, oxidative stress, and VEGF in the vitreous body

**Published:** 2010-01-29

**Authors:** Hiroshi Izuta, Nozomu Matsunaga, Masamitsu Shimazawa, Tetsuya Sugiyama, Tsunehiko Ikeda, Hideaki Hara

**Affiliations:** 1 Department of Biofunctional Evaluation, Molecular Pharmacology, Gifu Pharmaceutical University, Gifu, Japan; 2 Department of Ophthalmology, Osaka Medical College, Takatsuki, Japan

## Abstract

**Purpose:**

To investigate the relationships among antioxidant activities, oxidative stress, and vascular endothelial growth factor (VEGF) in the vitreous body and serum from proliferative diabetic retinopathy (PDR) patients.

**Methods:**

In 21 patients with PDR and 21 controls with macular hole (MH), the VEGF and lipid peroxide (Nε-hexanoyl-lysine [HEL]) levels in the vitreous and serum were measured by enzyme-linked immunosorbent assay, while antioxidant capacity (potential antioxidant [PAO]) was measured by chemical reduction of Cu^2+^.

**Results:**

Both the PAO and HEL levels in the vitreous and serum were significantly higher in PDR patients than in those with MH (both p<0.01). The VEGF concentrations in the vitreous were higher in PDR patients than in those with MH (p<0.01); however, the VEGF concentrations in the serum were not different between the two groups (p=0.95). Positive correlations were found between the PAO and VEGF concentrations and between the HEL and VEGF concentrations in the vitreous of both the PDR and the MH patients.

**Conclusions:**

Our study revealed that the PAO, HEL, and VEGF concentrations in the vitreous were increased in PDR versus MH patients and that there were positive correlations among these factors. This is consistent with VEGF and lipid peroxide levels in the vitreous playing some role in the pathogenesis of PDR.

## Introduction

Proliferative diabetic retinopathy (PDR), one of the complications of diabetes, is characterized by extensive neovascularization and vessel intrusion into the vitreous body, resulting in subsequent bleeding surrounding new vessels and leading to severe visual impairment. This process depends on the local production of angiogenic factors, such as vascular endothelial growth factor (VEGF), which potently activates angiogenesis, enhances collateral vessel formation, and increases permeability of the microvasculature [1]. VEGF expression, which is induced by high glucose levels and hypoxia, plays important roles in both normal and abnormal angiogenesis [2,3]. Its levels in the vitreous and aqueous body of the eye have been found to be markedly increased in patients with PDR [4,5]. Moreover, several clinical studies have shown that increased concentrations of VEGF within the eye have a strong correlation to the development of PDR [6].

The hyperglycemia-induced endothelial dysfunction that occurs in diabetes is not fully understood, but oxidative stress has been reported to play a key role in the initial insult. Multiple biochemical pathways that are known to increase the production of reactive oxygen species (ROS) have been linked to hyperglycemia/diabetes-induced vascular injury. These pathways include glucose auto-oxidation, the polyol pathway, and the formation of advanced glycation endproducts [7,8]. The tissues exposed to oxidative stress in diabetes include renal and ocular tissues, which tend to suffer damage and cause some of the complications of diabetes [9,10].

Oxygen free radicals can generate lipid peroxides, which are the products of oxidative fatty acid [11]. Nε-hexanoyl-lysine (HEL), an early stage of lipid peroxide, is formed from a lipid hydroperoxide and a lysine residue. HEL is considered to be one of the stable oxidative stress markers for lipid peroxide and protein modification [12]. A previous report indicated that HEL production is promoted by oxidatively modified low-density lipoprotein in rabbit atherosclerotic lesions [13]. Moreover, the plasma concentrations of HEL are increased in patients with azoospermia and oligospermia [14].

Oxidative stress is caused by the imbalance between ROS generation and antioxidant capacity. Therefore, for the accurate assessment of oxidative stress, it is important to measure both components. The use of potential antioxidant (PAO) is one of the approaches for evaluating antioxidant capacity; in this method Cu^2+^ is reduced by various antioxidants to Cu^+^, which is measured by colorimetry. PAO enables the evaluation of not only hydrophilic antioxidants, such as vitamin C and glutathione, but also hydrophobic antioxidants, such as vitamin E. This method also provides a quantitative measurement of the antioxidant capability of various biologic fluids, such as plasma and serum [14,15]. Therefore, PAO is a useful tool for clinical study to determine total antioxidant properties.

ROS is an angiogenesis inducer [16], and oxidative stress is believed to play a pivotal role in the development of diabetic retinopathy. However, relatively little information is available on antioxidant capacity and oxidative stress in PDR patients. In the present study, we aimed to clarify antioxidant capacity and oxidative stress in the vitreous body from PDR patients. We measured the following parameters in the vitreous and serum obtained from PDR and macular hole (MH) patients: (i) the levels of PAO by evaluating Cu^2+^ reduction by all antioxidants present, (ii) the concentrations of HEL (a marker of early stage lipid peroxide), and (iii) the concentrations of VEGF. We also designed the present study (a) to evaluate the relationship between antioxidant activity and oxidative stress in the vitreous body and serum obtained from PDR patients and (b) to determine whether the concentrations of VEGF correlate with other parameters.

## Methods

### Subjects and sample collection

This study was conducted according to the tenets of the Declaration of Helsinki and was performed after receiving approval from the institutional review committee of Osaka Medical College located in Osaka, Japan. Written informed consent was obtained from all patients after an explanation of the purpose of the study and the procedures involved.

The diabetic patients with PDR (10 men, 11 women) had an average age of 54.1 years (standard deviation [SD] ±13.6), while the average age of the patients with nondiabetic MH (7 men, 14 women) was 63.1±5.7 years. Undiluted vitreous samples were collected from 42 eyes of 42 individuals (PDR, 21 eyes; MH, 21 eyes) undergoing pars plana vitrectomy for the treatment of diabetic retinopathy and other retinal disorders at Osaka Medical College Hospital. Samples with repeat vitrectomy were excluded. We excluded the persons who had undergone repeated vitrectomy, and after the judgment, we used in the experiments (vitreous samples: PDR, 21 eyes; MH, 21 eyes, serum samples: PDR, 17 patients; MH, 18 patients). imultaneously, serum samples were collected from 35 individuals (PDR, 17 patients; MH, 18 patients). PDR patients with or without macular edema and traction membrane were included. MH patients without neovascular disease and vitreous hemorrhage were used as the controls for nondiabetic ocular disease because MH is caused by vitreomacular traction and we assumed that the vitreous body from patients with MH would be the most similar in constitution to normal eyes. Details of the patients with PDR and MH are shown in Table 1.

**Table 1:** Data for patients with proliferative diabetic retinopathy or macular hole .

**Characteristic**	**Macular hole (21 patients)**	**Proliferative diabetic retinopathy (21 patients)**
Age (years)	63.1±5.7	54.1±13.6
Number of women	14	11
**Clinical findings**		
Vitreous hemorrhage	−	21
+Traction membrane	−	12
+Cystoid macular edema	−	6
MH Stage 2	1	−
MH Stage 3	14	−
MH Stage 4	6	−

The table indicates the baseline characteristics and the complications of proliferative diabetic retinopathy and macular hole patients. Complications of each patient are shown in “clinical findings.” In proliferative diabetic retinopathy, majority of complications were vitreous hemorrhage. In macular hole patients, 1 patient was macular hole stage2, 14 patients were macular hole stage3, and 6 patients were macular hole stage 4. “Age” data are mean±SD.

Before intraocular infusion of a balanced salt solution, the vitreous core was cut and aspirated via the pars plana, with a vitreous cutter. The samples of vitreous bodies (0.6–0.8 ml) were spun for 10 min at 15,000× g in a refrigerated centrifuge at 4 °C to remove particles and then were stored in aliquots in polypropylene tubes at −80 °C until assay. At the same time as vitreous surgery, the samples of serum (2.0 ml) were collected in sterile tubes and rapidly frozen at −80 °C.

### Measurement of potential antioxidant levels

The evaluation of antioxidant capacity in vitreous and serum samples was performed by means of a PAO test (Nikken Seil Co., Ltd, Shizuoka, Japan). This method provides a quantitative measurement of the antioxidant capability of a biologic fluid, such as serum and plasma [14,15]. The antioxidant capability of our samples was obtained by evaluating Cu^+^ derived by the reduction of Cu^2+^ (Nikken Seil Co., Ltd., Shizuoka, Japan), which was added at known concentrations either to standard or to experimental samples. Cu^+^ forms a stable complex with bathocuproine (2,9-dimethyl-4,7-diphenyl-1,10-phenanthroline; Nikken Seil Co.), a colorimetric reagent of Cu^+^, and the complex has a typical absorption at 480–490 nm. The absorbance was measured using a microplate reader (Varioskan Flash TOP/BOTTOM; Thermo Fisher Scientific Inc., Waltham, MA).

### Measurement of Nε-hexanoyl-lysine levels

HEL is a lipid hydroperoxide-modified lysine residue that is considered to be a useful marker of early lipid peroxidation-derived protein modification [17]. HEL levels were determined with a competitive enzyme-linked immunosorbent assay (ELISA) kit (Nikken Seil Co., Ltd), which is used for quantitative measurement of hexanoyl lysine adducts. Using the ELISA method, minute concentrations of HEL can be detected. Methods were performed according to the manufacturer’s recommendations.

Briefly, microtiter plates (Nunc, Roskilde, Denmark) were coated with sample (10 mg protein/ml diluted with PBS). The coating solution was discarded, and the wells were washed three times with PBS containing 0.05% Tween-20 (TPBS; Bio-Rad, Tokyo, Japan), followed by distilled water. Each well was incubated with 100 ml of monoclonal antibody (Nikken Seil Co., Ltd.) against HEL for 2 h at 37 °Cwith shaking. After being washed with TPBS and distilled water, the wells were incubated for 1 h at 37 °C with 100 ml of peroxidase-labeled goat anti-mouse IgG (American Qualex, La Mirada, CA) diluted 1:5000 in TPBS. After another wash, 100 ml of o-phenylenediamine solution [5 mg of o-phenylenediamine (ICN Pharmaceuticals, Inc., Mesa, CA) and 10 ml of 30% H_2_O_2_ (Wako Pure Chemical Industries, Osaka, Japan) in 10 ml of 0.1 M citrate phosphate buffer, pH 5.5 (Polysciences, Inc., Warrington, PA)] was added to each well. The plates were incubated for 15 min at room temperature. Adding 50 ml of 2N sulfuric acid (Wako) terminated the reaction. Absorbance at 492 nm was read using a microplate reader (VarioskanR Flash, Thermo Electron Corporation, Vantaa, Finland).

### Measurement of vascular endothelial growth factor concentrations

The VEGF concentrations in vitreous body and serum were measured using a human VEGF ELISA kit (Pierce Biotechnology, Rockford, IL). In brief, 50 ml of sample diluent and 50 ml of either the standard control or a fivefold diluted vitreous body or serum sample were added to each well of the ELISA plate, incubated for 2 h at room temperature, and washed three times at room temperature (Endogen VEGF ELISA Kit). Then, 100 ml of anti-human VEGF biotinylated antibody reagent (Endogen VEGF ELISA Kit) was added to each well, incubated for 1 h at room temperature, and washed three times at room temperature. Streptavidin- horseradish peroxidase (HRP) reagent (Endogen VEGF ELISA Kit) was added to each well, incubated for 30 min at room temperature, and then washed three times at room temperature. Next, 100 ml TMB substrate solution (Endogen VEGF ELISA Kit) was added to each well, and the plate was developed in darkness at room temperature for 30 min. Finally, 100 ml of stop solution was added, and concentrations were determined at 450 nm (correction 550 nm) using a microplate reader.” Further information is not clear, because the details are not described in instruction of “Endogen VEGF ELISA Kit.

### Statistical analysis

Statistical analysis was performed with the aid of the Statistical Package for the Social Sciences 15.0J for Windows software (SPSS Japan Inc., Tokyo, Japan). Data are presented as mean±SD. Statistical comparisons were made using the Kruskal–Wallis test, followed by the Tukey test. To examine correlations, the Pearson's product-moment correlation coefficient was used. A value of p<0.05 was considered to indicate statistical significance.

## Results

### Potential antioxidant levels

**Figure 1:**
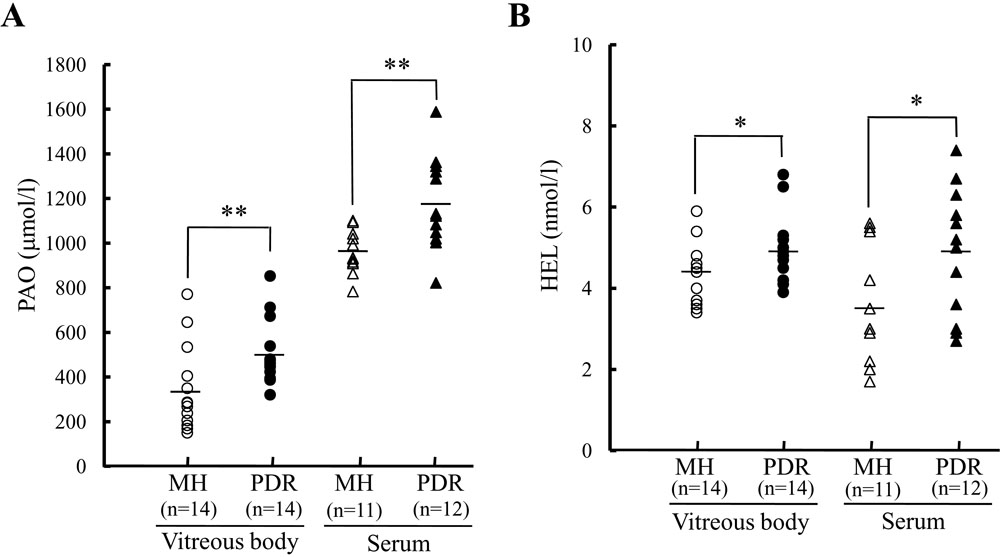
Potential antioxidant and Nε-hexanoyl-lysine levels in proliferative diabetic retinopathy and macular hole patients. The vitreous and serum levels of (**A**) potential antioxidant (PAO) and (**B**) Nε-hexanoyl-lysine (HEL) were significantly higher in proliferative diabetic retinopathy (PDR) versus macular hole (MH). *p<0.05, **p<0.01 versus MH (Tukey test).

As shown in Figure 1A, the PAO levels in the vitreous and serum were significantly higher (p<0.01) in the PDR patients (505.0±145.0 μmol/l and 1177.9±207.8 μmol/l, respectively) than in the MH patients (331.7±193.5 μmol/l and 962.5±97.6 μmol/l, respectively). Repeated within-run and between-run assay variabilities were always lower than 5%.

### Nε-hexanoyl-lysine levels

As shown in Figure 1B, the levels of HEL in the vitreous and serum were significantly higher (p<0.05) in PDR patients (4.9±0.8 nmol/l and 4.9±1.6 nmol/l, respectively) than in MH patients (4.3±0.7 nmol/l and 3.5±1.4 nmol/l, respectively).

### Relationship between potential antioxidant and Nε-hexanoyl-lysine levels

**Figure 2:**
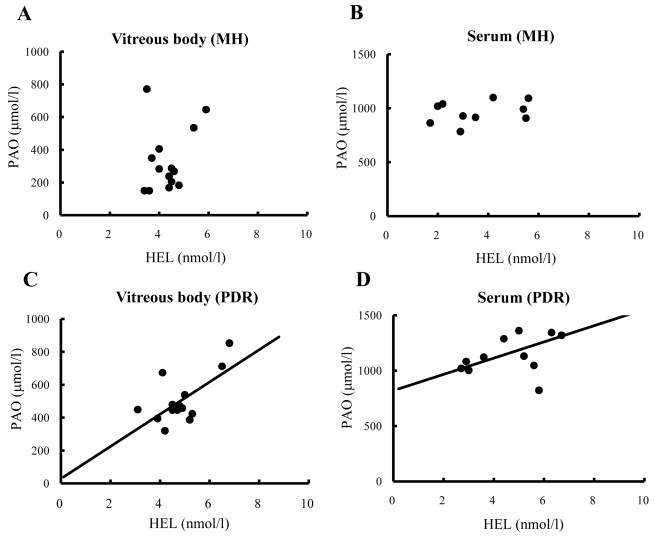
Relationships between potential antioxidant and Nε-hexanoyl-lysine in the vitreous body and serum from proliferative diabetic retinopathy and macular hole patients. In the vitreous body (**A**) and serum (**B**), no significant correlations were evident between PAO and HEL levels in MH patients. **C**: There was a positive correlation between PAO and HEL levels in the vitreous body (r=0.65, p<0.01) in PDR patients. **D**: The concentrations of PAO and HEL in the serum showed a positive correlation (r=0.55, p<0.05) in PDR patients. Correlations were examined using the Pearson's product-moment correlation coefficient.

In the PDR patients, there was a significant positive correlation between PAO and HEL levels both in the vitreous body (r=0.65, p<0.01) and the serum (r=0.55, p<0.05; Figure 2C,D). On the other hand, there was no significant correlation between PAO and HEL levels either in the vitreous body (r=0.27, p=0.17) or the serum (r=0.32, p=0.17) obtained from the MH patients (Figure 2A,B).

### Vascular endothelial growth factor concentrations

**Figure 3:**
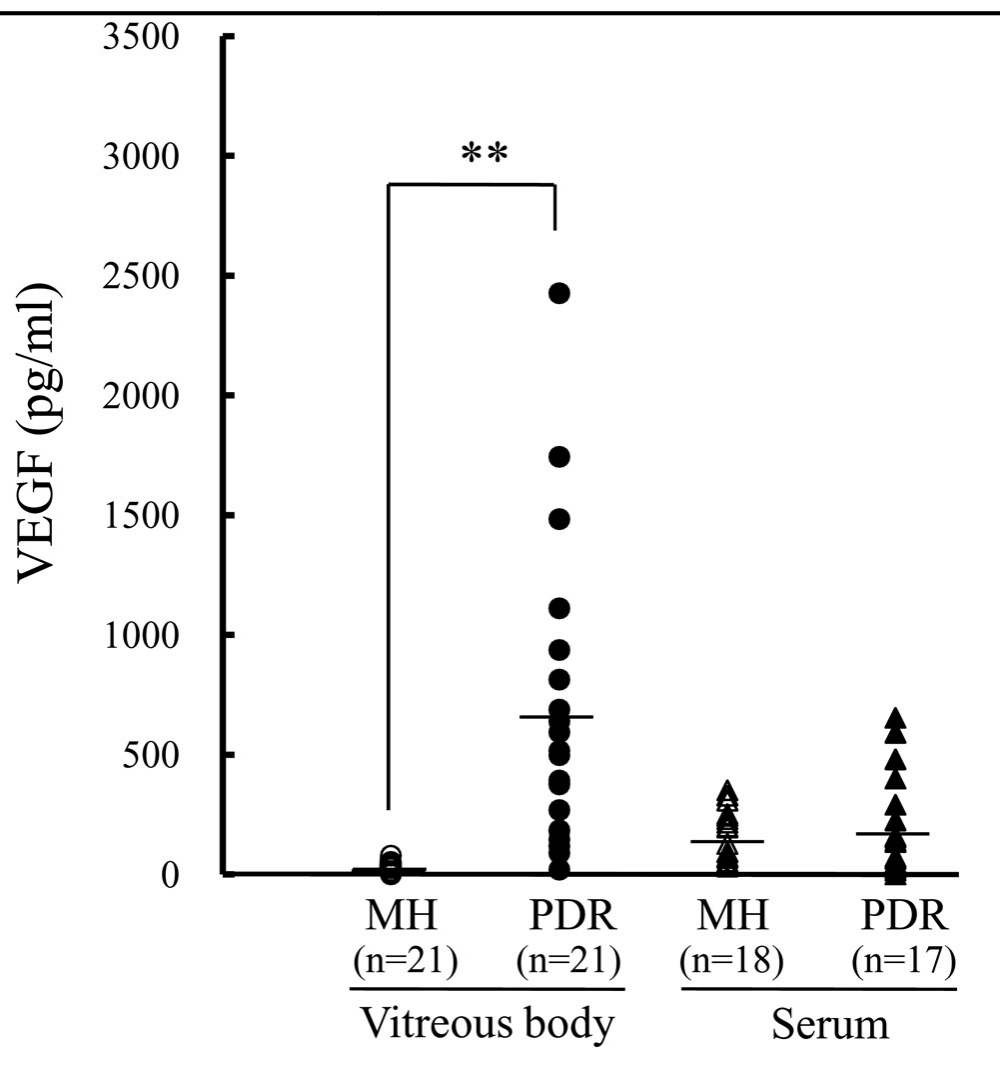
Vascular endothelial growth factor concentrations in the vitreous body and serum from proliferative diabetic retinopathy and macular hole patients. The VEGF concentrations in the vitreous were much higher in PDR than in MH patients. In contrast, the VEGF concentrations in the serum were not significantly different between PDR and MH patients. **, p<0.01 versus MH (vitreous body; Tukey test).

As shown in Figure 3, the concentrations of VEGF in the vitreous were strikingly higher (p<0.01) in the PDR (598.8±604.5 pg/ml) than in the MH (30.9±21.9 pg/ml) patients. In contrast, the concentrations of VEGF in the serum were not significantly different between the two patient groups (PDR, 253.4±229.3 pg/ml; MH, 199.6±111.2 pg/ml; p=0.95).

### Relationships between vascular endothelial growth factor and potential antioxidant levels and between vascular endothelial growth factor and Nε-hexanoyl-lysine levels

**Figure 4:**
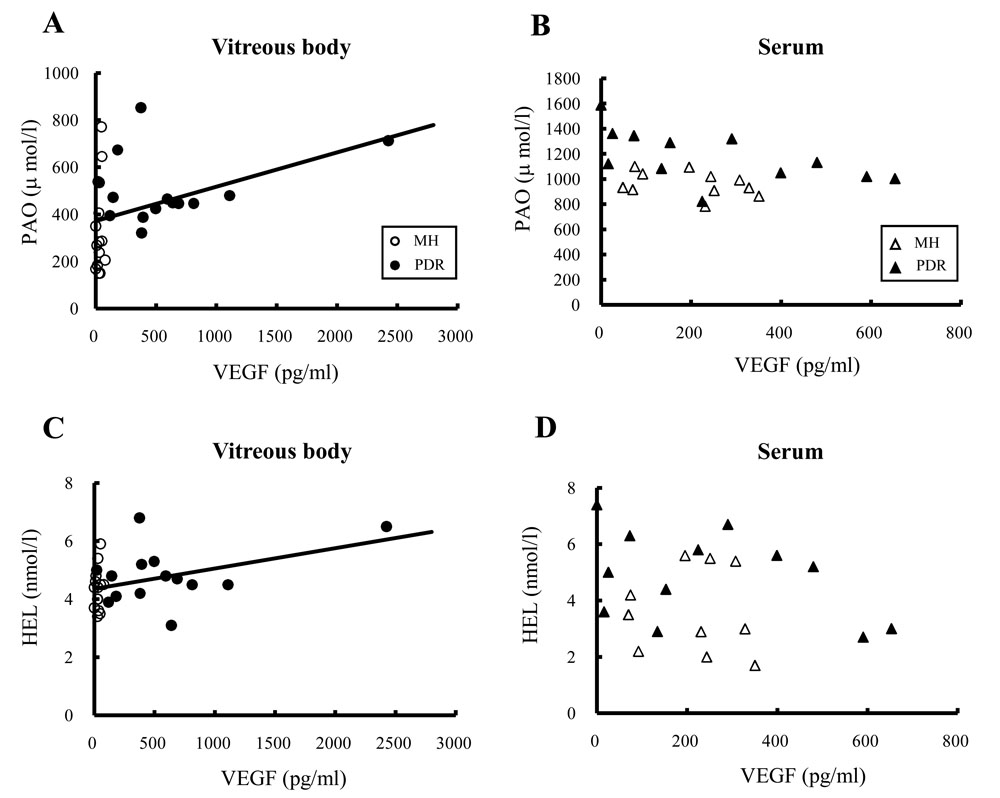
Relationships between potential antioxidant and vascular endothelial growth factor and between Nε-hexanoyl-lysine and vascular endothelial growth factor in the vitreous body and serum from proliferative diabetic retinopathy and macular hole patients. **A**: The vitreous concentrations of PAO and VEGF displayed a significant correlation (r=0.39, p<0.05). **B**: In the serum, there was no significant correlation between PAO and VEGF concentrations. **C**: The HEL and VEGF concentrations in the vitreous showed a significant correlation (r=0.42, p<0.05). **D**: In the serum, there was no significant correlation between HEL and VEGF concentrations. Open circles and closed circles indicate MH and PDR patients, respectively (vitreous body). Open triangles and closed triangles indicate MH and PDR patients, respectively (serum). Correlations were examined using the Pearson's product-moment correlation coefficient.

We performed association-based analysis to examine possible relationships between VEGF and PAO levels and between VEGF and HEL levels in the vitreous and the serum samples (Figure 4). In the vitreous body, VEGF concentrations were positively correlated with PAO (r=0.39, p<0.05) and HEL (r=0.42, p<0.05) levels in all patients (Figure 4A,C). However, in the serum, VEGF concentrations were not significantly correlated with PAO (r=-0.35, p=0.10) or HEL (r=-0.23, p=0.31) levels (Figure 4B,D).

## Discussion

In the present study, we investigated the concentrations of PAO, HEL, and VEGF in vitreous and serum samples taken from PDR and MH patients. The PAO and HEL concentrations in both the vitreous and serum were significantly higher in the PDR patients than in the MH patients. We found a positive correlation between PAO and HEL levels both in the vitreous and the serum obtained from the PDR patients. In addition, the VEGF concentrations in the vitreous were higher in the PDR than in the MH patients, and there was a positive correlation between VEGF and PAO levels and between VEGF and HEL levels in the vitreous body from all patients. This is the first time that the novel lipid peroxide HEL has been measured in the vitreous body from PDR patients.

Retinal neovascularization is one pathogenesis of PDR. Previous reports have indicated that neovascularization is well correlated with VEGF concentrations in the vitreous but not in the serum [4,5], which is consistent with the results of our study. It is unclear why the vitreous VEGF concentrations of PDR patients are more than 20 times higher than concentrations in the nondiabetic controls (MH patients), but it is certain that the upregulation of VEGF in the vitreous is an important factor in the pathogenesis of PDR [6].

Over the past decade there has been substantial interest in oxidative stress and its potential roles in diabetogenesis, development of diabetic complications, atherosclerosis, and associated cardiovascular disease. Consequences of oxidative stress lead to damage of DNA, proteins, and lipids, disruption in cellular homeostasis, and accumulation of damaged molecules. Previous reports showed that levels of the lipid peroxidative products 4-hydroxynonenal and malondialdehyde in the vitreous and serum are significantly higher in patients with diabetic retinopathy than in nondiabetic controls [16]. In the present study, an increase in HEL concentrations was observed in the vitreous bodies obtained from PDR patients, suggesting that oxidative stress (such as ROS generation) may lead to cellular damage and to disease development and pathogenesis in PDR patients.

ROS generation also plays a pivotal role against angiogenesis via upregulation of VEGF expression both in vitro and in vivo [18]. Colavitti et al. reported that ROS is one of the downstream mediators of the VEGF signaling pathway [19], while Ushio-Fukai and colleagues noted that the angiogenic effects of ROS are attenuated by *N*-acetylcysteine, a metabolic precursor of reduced glutathione, in endothelial cells [20]. Hence, ROS may perform pivotal roles in various angiogenic events. In the present study, an increase in lipid peroxide (as represented by HEL) and VEGF levels were observed in the vitreous bodies obtained from PDR patients, and there was a significant correlation between HEL and VEGF concentrations in the vitreous bodies from both groups of patients. These results indicate that oxidative stress, such as from increased ROS generation, may play a pivotal role in the upregulation of VEGF in the vitreous body in PDR patients.

In our study, the intravital antioxidant capacity was increased during a state of enhanced oxidative stress in an advanced stage of diabetic retinopathy; however, some reports have indicated that antioxidant capacity is decreased in PDR patients [21,22]. This discrepancy in antioxidant capacity may be a result of focusing on different types of antioxidant enzymes. Although catalase and glutathione are decreased in PDR patients [21,22], extracellular SOD is upregulated in PDR patients [23]. These results indicate that the increased antioxidant capacity in the present study may result from the summation of increased and decreased antioxidant capacity of various enzymes. However, further investigations are needed to clarify the antioxidant capacity of individual enzymes in PDR patients.

ROS are products of normal cellular metabolism and are known to act as second messengers. Under pathological conditions, ROS generation in various diseases is accompanied by activation of redox homeostasis to protect cells against oxidative stress. In our study the total antioxidant capacity was increased with accompanying upregulation of HEL in the vitreous body from PDR patient. Understanding how the redox regulatory system increase the antioxidant capacity in the vitreous bodies in PDR patients. Redox regulation is essential machinery to maintain homeostasis of redox state. Major factors to regulate redox state are glutathione, thioredoxin, and nuclear factor erythroid-2-related factor 2 (Nrf2) pathway [24]. The glutathione antioxidant system is one of the redox regulations. Nicotinamide adenine dinucleotide phosphate is required to maintain the glutathione cycles, but in diabetes mellitus it is known that activation of the polyol pathway leads to depletion of NADPH, which results in the antioxidant capacity of glutathione being decreased in diabetes patients [25]. A second redox regulation is thioredoxin. A previous study indicated that thioredoxin levels are significantly higher in diabetic patients than in healthy controls [26]. A third candidate is nuclear factor erythroid-2-related factor 2 (Nrf2). Nrf2 is a redox-sensitive transcription factor that binds to the antioxidant response element (ARE) in the promoter region of phase II detoxifying and antioxidant enzymes, leading to an upregulation of antioxidant gene expression in vascular cells. In diabetes patients *glutathione-s-transferase*, one of the downstream genes regulated by the Nrf2/ARE pathway, is upregulated [25]. Moreover, hyperglycemia-induced ROS production is exacerbated in Nrf2 knockout mice [27,28], further implicating the Nrf2/ARE pathway in the defense against oxidative stress in diabetic complications. These findings indicate that the thioredoxin and Nrf2/ARE pathways are candidates for redox regulation to maintain the redox status in the vitreous body from PDR patients. However, further examination is needed to clarify the relationship between PDR and the redox regulation.

In summary, our results reveal that lipid peroxides and antioxidant activities were increased in the vitreous body and serum samples obtained from PDR patients and there were positive correlations among these factors. Moreover, increased VEGF concentrations were significantly correlated with lipid peroxide concentrations and antioxidant activities in the vitreous body from PDR patients. These results suggest that lipid peroxides and antioxidants to involved in the pathogenesis of PDR.
